# Protocol for a randomized controlled trial of Xueshuantong injection on myocardial injury and residual cardiovascular risk in patients with unstable angina

**DOI:** 10.3389/fcvm.2025.1602666

**Published:** 2025-10-21

**Authors:** Yaxing Wang, Guangyu Liu, Zhenkai Lu, Junyu Xi, Wenye Feng, Yutong Ma, Jian Lyu, Yanming Xie

**Affiliations:** ^1^ Xiyuan Hospital, China Academy of Chinese Medical Sciences, Beijing, China; ^2^ Institute of Basic Research in Clinical Medicine, China Academy of Chinese Medical Sciences, Beijing, China; ^3^ Wangjing Hospital, China Academy of Chinese Medical Sciences, Beijing, China; ^4^ Key Laboratory of Chinese Internal Medicine of Ministry of Education, Dongzhimen Hospital, Beijing University of Chinese Medicine, Beijing, China

**Keywords:** unstable angina, myocardial injury, cardiovascular risk, Xueshuantong for injection, randomized controlled trail

## Abstract

**Background:**

Unstable angina (UA) is a critical subtype of acute coronary syndrome (ACS). Myocardial injury is a key determinant of disease progression and long-term prognosis, yet it often persists despite standard therapy. In addition, residual inflammation remains an important risk factor for adverse outcomes. Xueshuantong Injection Lyophilized (XST), derived from *Panax notoginseng* saponins (PNS), has shown potential to reduce myocardial injury and modulate inflammatory responses in cardiovascular disease, but its efficacy in UA has not been fully evaluated.

**Methods:**

This is a randomized, parallel control, double-blind, small-sample exploratory clinical trail. Participants will be recruited from Xiyuan Hospital, China Academy of Chinese Medical Sciences (Beijing, China). Eligible patients with UA will be randomized into two groups. The intervention group will receive XST 500 mg intravenously once daily for 7 days, and the control group will receive XST 25 mg intravenously once daily for 7 days. The primary outcome is CK-MB at Day 7. Secondary outcomes are cTnT, NT-proBNP, inflammatory/endothelial biomarkers (hs-CRP, IL-6, MMP-9, VEGF, HMGB1), and angina-related parameters (attack frequency, symptom severity).

**Ethics and Registration:**

The trial has been approved by the Ethics Committee of Xiyuan Hospital and registered in the ITMCTR on March 21, 2025, http://itmctr.ccebtcm.org.cn (No. ITMCTR2025000552).

**Conclusion:**

This exploratory study will evaluate the efficacy and safety of XST in reducing myocardial injury and residual risk in UA patients, providing evidence for future large-scale confirmatory trials.

## Introduction

Unstable angina (UA), a critical subtype of acute coronary syndrome (ACS), represents a major acute cardiovascular event alongside myocardial infarction (MI). Cardiovascular diseases (CVDs) collectively account for represent approximately 30% of global mortality, with ACS remaining a predominant contributor to cardiovascular-related deaths. Without timely intervention, UA carries a high risk of progression to MI, potentially leading to severe complications or mortality (1–3). In China, the burden of CVD continues to rise, with mortality rates attributable to CVD reaching 46.74% in rural and 44.26% in urban populations, exerting substancial pressure on healthcare infrastructure (4). Although contemporary revascularization strategies and pharmacotherapies [including percutaneous coronary intervention (PCI) and optimized antiplatelet regimens] have improved outcomes, patients remain at high risk for recurrent cardiovascular events. Current therapeutic paradigms prioritize antiplatelet agents and lipid-lowering strategies but lack targeted interventions to MI and residual inflammatory-metabolic risk. Despite advances in PCI and pharmacotherapy, 20%–30% of UA patients still experience perioperative myocardial injury—a subclinical entity defined by elevated high-sensitivity cardiac troponin (hs-cTn) levels—that independently predicts adverse outcomes (5). Recent evidence further demonstrates that elevated cardiac troponin I and B-type natriuretic peptide (BNP) levels independently predict major adverse cardiovascular events (MACE) and all-cause mortality in hemodialysis patients, with synergistic risk stratification when combined (6).

Xueshuantong Injection Lyophilized (XST) is a standardized traditional Chinese medicine (TCM) formulation primarily composed of *Panax notoginseng* saponins (PNS). With documented therapeutic effects in promoting blood circulation, resolving stasis, and improving vascular patency, XST is clinically used as an adjuvant therapy for ACS (7). Modern pharmacological studies have elucidated its neuroprotection mechanisms, including anti-inflammatory actions through suppression of PI3K-Akt signaling and endothelial function enhancement via improved nitric oxide bioavailability, offering multi-target modulation of ACS pathophysiology (8–11).

We previous conducted a multicenter, clinical trail (Registration No. ChiCTR1800015911) in provinces/municipalities in China from 2020 to 2022 (12). The aim of this study was to evaluate the efficacy and safety of XST in combination with dual antiplatelet therapy on MACE in patients with UA. A total of 1229 UA patients from 16 provincial administrative regions in China were enrolled in the study. The results of the median follow-up of 6 months showed that the incidence of MACE was reduced by 46.7% in the XST group compared with the control group, with time to MACE onset prolonged (27.88 vs. 19.60 days), and no significant difference in major bleeding events between the two groups. Although this trial confirmed that XST significantly reduced MACE risk, the underlying mechanisms remained unclear. Based on this, we designed the present study to test the hypothesis that XST may exert cardiovascular protection by attenuating myocardial injury and ameliorating residual inflammatory risk, as assessed by biomarkers including CK-MB, cTnT, NT-proBNP, hs-CRP, IL-6, MMP-9, VEGF, and HMGB1. This trial aims to elucidate the pleiotropic cardioprotective mechanisms of XST and provide evidence for its integration into precision secondary prevention strategies.

## Methods and analyses

### Study design

This randomized, double-blind, parallel-controlled, exploratory clinical trial (Registration NO. ITMCTR2025000552) was prospectively registered prior to patient enrollment. Participants will be recruited from Xiyuan Hospital, China Academy of Chinese Medical Sciences (Beijing, China), and all trial data will be collected from this cohort. The study is designed evaluate XST as an adjunctive therapy for patients with UA, with a primary focus on its potential to attenuate myocardial injury and reduce residual cardiovascular risk by modulating microvascular thrombosis and chronic inflammation. The findings are expected to provide evidence supporting the cardioprotective role of XST and its potential integration into precision-oriented secondary prevention strategies.

**Figure 1:**
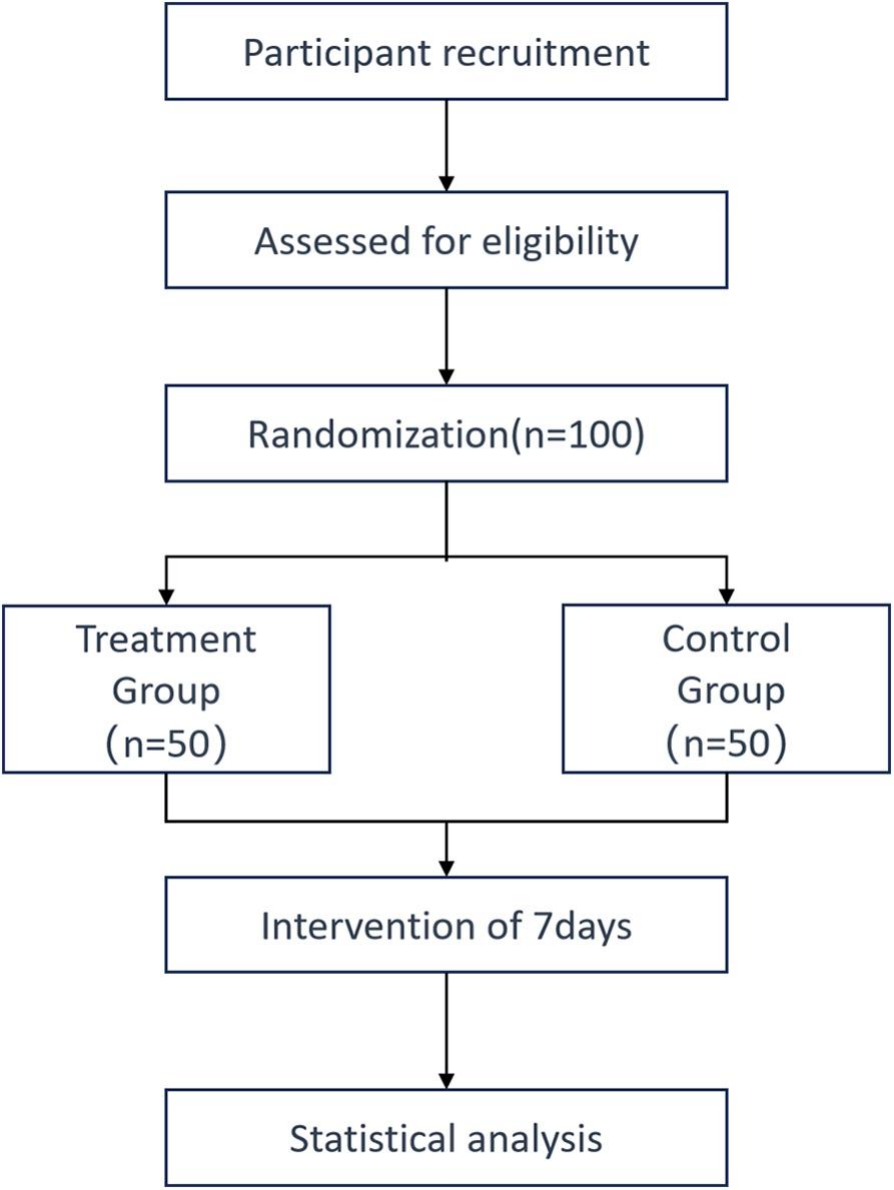
The flow chart of the trial.

Eligible participants will be randomized into two groups: the treatment group will receive XST 500 mg, while the control group will receive XST 25 mg. Written informed consent (see online supplemental material) will be obtained from all participants or their legal representatives after eligibility screening and before randomization. A detailed flowchart of the study protocol is provided in Figure 1.

## Participants

### Diagnostic criteria

The diagnosis of UA was made in accordance with the 2019 European Society of Cardiology (ESC) Guidelines for the diagnosis and management of chronic coronary syndromes (13) Diagnostic criteria included frequent anginal episodes, ST-segment depression 0.1 mV in two or more contiguous leads, and/or symmetrical T-wave inversion. Diagnostic confirmation was based on findings from echocardiography and myocardial enzyme analysis.

The diagnosis of cardiovascular blood stasis syndrome was based on the formulation principles and indications of the lyophilized XST injection, and referenced the 2018 Diagnostic Criteria for Evidence Elements of Coronary Heart Disease Angina Pectoris established by the Cardiovascular Disease Branch of the Chinese Medical Association (14). Primary symptom: stabbing chest pain with fixed location; Secondary symptoms: chest tightness, palpitations, fatigue, dark complexion; Tongue and pulse: dark purple tongue with petechiae or ecchymosis, thin coating; pulse choppy or intermittent. Diagnosis requires the primary symptom plus at least one secondary symptom, in combination with tongue and pulse findings.

### Inclusion criteria

Patients meeting all of the following conditions will be eligible for enrollment in this study:

1. Diagnosis of coronary artery disease (CAD): Fulfillment of the diagnosis of CAD diagnosis with at least one of the following criteria: (1) Definite history of myocardial infarction; (2) Previous coronary artery revascularization (PCI) (3) Coronary angiography demonstrating ≥50% luminal stenosis in at least one major coronary artery; (4) Evidence of myocardial ischemia confirmed by cardiac magnetic resonance imaging or nuclear myocardial perfusion imaging.
2. Diagnosis of UA: Meeting the diagnostic criteria for UA and at least one of the following: (1) Electrocardiographic evidence of transient or persistent ST-segment deviation ≥0.1 mV in one or more leads; (2) TIMI risk score ≥3;
3. Traditional Chinese medicine (TCM) diagnosis: Diagnosis of cardiac blood stasis syndrome according to TCM criteria.
4. Age; 45–75 years old.
5. Informed consent: Hospitalized patients who voluntarily agree to participate, accept study medication, and sign a written informed consent form. The informed consent process will be conducted in accordance with the relevant provisions of Good Clinical Practice (GCP).

### Exclusion criteria

Patients meeting any of the following conditions will be excluded from this study:

1. Severe cardiovascular conditions: (1) New York Heart Association (NYHA) class IV heart failure; (2) UA with high short-term risk stratification; (3) Uncontrolled grade III hypertension (systolic blood pressure ≥180 mm Hg and/or diastolic blood pressure ≥110 mm Hg).
2. Increased risk of bleeding: History of major organ bleeding within the past six months; Thrombocytopenia or coagulation abnormalities; Active bleeding within the past month.
3. Severe comorbidities or contraindications: Abnormal liver or renal function tests; Pregnant or breastfeeding women, or those planning pregnancy during the study period; Patients with malignant tumors, severe psychiatric disorders, or other serious systemic diseases affecting survival.
4. Patients who refused to participate in this study.

### Randomization and blinding

This study used a central stratified block randomization method. We used statistical software to create two treatment groups (test and control) for 100 patients. Each patient from number 01 to 100 was randomly assigned to a group using a treatment allocation table. The blinding process was done by statisticians.

This was a double-blind study. The control group received a very low dose, and both groups had the same number of patients (1:1 ratio). The study drugs and control were made to look identical in packaging and appearance. The packaging followed the random allocation table and blinding rules. The sealed blinding codes were stored in two copies at the clinical trial institution.

### Interventions

All enrolled patients will receive guideline-recommended standard Western medical therapy, including dual antiplatelet therapy, statins, β-blockers, and ACEI/ARB, as appropriate. On this basis, patients will be randomly assigned to receive:

1. Treatment group: The patients will be treated via an intravenous drip with 500 mg XST diluted with 250–500 mL of 5% glucose injection or sodium chloride injection, once per day for 7days;
2. Control group: The patients will be treated via an intravenous drip with 25 mg XST diluted with 250–500 mL of 5% glucose injection or sodium chloride injection, once per day for 7days.

The choice of a low-dose control (25 mg/day) was made solely to preserve the integrity of blinding, as this minimal dose is expected to have negligible pharmacological effect and approximate placebo. This design ensured that both groups received visually identical interventions, minimizing the risk of unblinding among patients and nursing staff.

### Outcome measurements

Primary and secondary efficacy outcomes were measured once pre-treatment and once post-treatment for a total of two times.

### Primary outcomes

The primary efficacy endpoint of this study is the change from baseline in CK-MB at Day 7.

### Secondary outcomes

1. Change from baseline in cTnT and NT-proBNP at Day 7.
2. Inflammatory and endothelial biomarkers: changes from baseline to Day 7 in hs-CRP, IL-6, MMP-9, VEGF and HMGB1.
3. Angina-specific parameters: changes in attack frequency, symptom severity.

### Safety assessment

An adverse event (AE) refers to any unintended medical occurrence observed during the trial, regardless of its relationship to the study intervention. Throughout the study period, researchers will monitor vital signs (e.g., blood pressure, heart rate); Perform blood tests, urinalysis, and assess renal/liver function (at baseline and post-treatment). Document any clinically significant abnormalities or AEs identified during the trial.

If an AE occurs, the research team will clinically evaluate whether to pause participant involvement, initiate diagnostic procedures, or adjust treatment plans. For severe AEs (SAEs), immediate medical intervention will be prioritized to ensure participant safety. All SAEs must be reported to the institutional ethics committee within 24 h of identification.

### Data collection and management

In this experiment, an electronic data acquisition system was used to create an electronic case report form, and the data were collected and managed online through the Internet. After the blind review of the data is completed and locked, the blind is uncovered for the first time when the statistician conducts statistical analysis, and the corresponding processing group (i.e., group A and Group B) is opened. After the statistical analysis, the study report (discussion draft) was written by the main researcher, and the blind was uncovered for a second time, and the corresponding study group of the treatment group was selected.

### Sample size

The sample size was determined based on the sole primary endpoint (CK-MB). Based on previous reports of CK-MB changes in unstable angina patients (15), we conservatively assumed a standardized effect size of d = 0.50 (medium effect). With two-sided α = 0.05% and 80% power, the required sample size for a two-sample t-test was 63 per group. Because the primary analysis will use ANCOVA with baseline adjustment, the sample size was reduced by a factor of (1–R²), assuming R² = 0.40, yielding approximately 38 per group. After considering a 20% attrition rate, the final target enrollment was 50 per group, 100 in total. Calculations were performed using G*Power v3.1.9.7

### Analysis sets

#### Full analysis set (FAS)

Defined according to the intention-to-treat principle, including all randomized participants who received at least one dose of study medication and had at least one post-baseline efficacy assessment. The FAS will be used for the primary efficacy analysis.

#### Per-protocol set (PPS)

Includes participants who completed the study without major protocol deviations (e.g., non-adherence, prohibited concomitant medication, significant missing data). The PPS will be used for sensitivity analyses to evaluate the robustness of the results.

#### Safety set (SS)

Includes all participants who received at least one dose of study medication. The SS will be used for safety analyses.

### Statistical analysis

Statistical analyses will be performed using R software (version 4.3.2). All tests will be two-sided with α=0.05. The primary outcome (Day-7 CK-MB, baseline-adjusted) will be analyzed by ANCOVA, with treatment group as a fixed factor and baseline CK-MB as a covariate. Major concomitant medications will also be included as covariates, specifically: (1) dual antiplatelet therapy; (2) statins; (3) β-blockers; and (4) ACEI/ARB. For each class, exposure during the 7-day intervention window will be coded “Yes” if the drug was taken on ≥80% of on-study days (≥6/7 days), otherwise “No”. The treatment effect will be reported as the adjusted mean difference with 95% CI. Secondary outcomes will be analyzed using ANCOVA or appropriate parametric/non-parametric tests with the same covariates. *P*-values for these outcomes will be nominal without multiplicity adjustment and interpreted as exploratory. Safety outcomes will be summarized descriptively by incidence (*n*, %) and compared between groups using χ² or Fisher's exact test, as appropriate.

## Discussion

UA remains characterized by high residual cardiovascular risk, largely attributable to incomplete plaque healing and ongoing myocardial injury despite guideline-recommended therapy (16, 17). Previous large-scale multicenter studies with XST focused mainly on symptom relief or composite cardiovascular outcomes, but did not specifically evaluate myocardial injury, which is closely linked to both short- and long-term prognosis. Biomarkers such as CK-MB and cTnT therefore represent clinically meaningful endpoints for UA patients. The present trial adopts myocardial injury as the primary outcome to directly assess the cardioprotective potential of XST. In parallel, biomarkers of inflammation and endothelial dysfunction are included as secondary outcomes, allowing evaluation of XST's effect on residual risk dimensions. This dual-level design is intended to complement prior evidence and provide more comprehensive insights into clinical efficacy.

A growing body of preclinical evidence provides biological plausibility for the proposed trial. In ischemic models, XST inhibited shear-induced platelet aggregation through Piezo1–Ca²^+^ signaling, suggesting benefits in reducing thrombosis under high shear conditions (18). PNS improved endothelial function and microvascular repair via activation of the Nrf2–VEGF axis (19) and by promoting VEGF-mediated angiogenesis (20). Epigenetic modulation through miR-200a demethylation further supported angiogenic effects (21). In acute myocardial infarction, proteomic analyses indicated XST enhanced pathways of cellular survival and energy metabolism (22). PNS also attenuated myocardial fibrosis by upregulating miR-29c and modulated autophagy in ischemia–reperfusion settings (23, 24). Additional findings, including regulation of the AMPK–mTOR pathway in cerebral I/R injury, reinforce its multi-target protective actions (25). Collectively, these results suggest XST may mitigate multiple aspects of UA pathophysiology, including platelet activation, endothelial dysfunction, inflammation, oxidative stress, fibrosis, and maladaptive remodeling.

Preliminary clinical studies reported symptomatic and electrocardiographic improvements when XST was added to standard therapy in patients with dual antiplatelet resistance (26). A systematic review and meta-analysis indicated potential efficacy and good safety, although the methodological quality of included trials was limited (27). More recently, an expert consensus recommended XST as an adjunctive treatment for UA, with standardized indications and dosing guidance (28). Furthermore, a recent review summarized the pleiotropic cardiovascular effects of PNS preparations and emphasized the need for higher-quality randomized controlled trials (29). Together, these findings highlight both the therapeutic potential of XST and the need for rigorous confirmation in well-designed clinical studies.

Building on this evidence, the present trial employs a randomized, double-blind design with clearly defined endpoints. The choice of myocardial injury as the primary outcome, combined with inflammatory and endothelial markers as secondary outcomes, aims to generate more robust evidence on both cardioprotection and residual risk reduction. In addition, integrating TCM syndrome scores with modern biomarkers will provide a broader evaluation framework. If successful, this study may clarify the role of XST as an adjunctive therapy for UA and establish a foundation for future translational research linking clinical biomarkers with mechanistic targets such as Nrf2–VEGF, miR-29c, miR-200a, Piezo1, and autophagy-related pathways.
